# Vitex agnus castus effects on hyperprolactinaemia

**DOI:** 10.3389/fendo.2023.1269781

**Published:** 2023-11-21

**Authors:** Lídice Tavares Puglia, Jean Lowry, Gianluca Tamagno

**Affiliations:** ^1^ Department of Endocrinology/Diabetes Mellitus, Mater Misericordiae University Hospital, Dublin, Ireland; ^2^ Department of Medicine, Blackrock Clinic and Hermitage Clinic - Blackrock Health, Dublin, Ireland

**Keywords:** Vitex agnus castus, hyperprolactinaemia, dopamine agonist, phytotherapy, premenstrual syndrome, mastalgia

## Abstract

**Background:**

Vitex agnus castus (VAC), also known as chaste tree, is a plant from the Mediterranean area, Crimea, and central Asia. Its fruit has been used for more than 2500 years as phytotherapic agent. In the last century, VAC has been mostly used for the treatment of premenstrual syndrome (PMS), menstrual irregularities, fertility disorders, and symptoms of menopause. Since some degree of hyperprolactinaemia may be observed in patients with such disorders, VAC effects on hyperprolactinaemia have been assessed in a small number of studies and in some patient series or single case reports. It has been postulated that the diterpenes contained in VAC extract may interact with dopamine D2 receptors (D2R) and inhibit prolactin release via dopamine D2R activation in the anterior pituitary. Most of the published papers focus on the use of VAC for the management of PMS or infertility. However, due to its action on D2R, VAC could have a role in the treatment of mild hyperprolactinaemia, including patients with idiopathic hyperprolactinaemia, microprolactinoma, drug-induced hyperprolactinaemia, or polycystic ovary syndrome.

**Methods:**

We have reviewed and analysed the data from the literature concerning the use of VAC extracts in patients with hyperprolactinaemia.

**Results:**

Some evidence suggests a possible role of VAC for the management of hyperprolactinaemia in selected patients, though in an inhomogeneous way. However, there are not any large randomized controlled trials supporting the same and the precise pharmacological aspects of VAC extract in such a clinical setting still remain obscure.

**Conclusion:**

It appears that VAC may represent a potentially useful and safe phytotherapic option for the management of selected patients with mild hyperprolactinaemia who wish to be treated with phytotherapy. However, larger studies of high quality are needed to corroborate it.

## Introduction

### Rationale

The genus Vitex comprises nearly 250 species distributed worldwide, mostly in the tropical and temperate zones (1). Several species, such as Vitex agnus castus (VAC), Vitex trifolia, Vitex negundo, and Vitex rotundifolia have been used as traditional medicinal plants. More than 20 Vitex species have been investigated with regard to their chemical and biological properties with nearly 200 compounds described so far (2).

VAC lamiaceae, formerly verbenaceae, is a deciduous tree or a large shrub native to central Asia and Mediterranean Europe. VAC is also known as chaste tree, chaste berry, or monk’s pepper. The dried fruits of VAC have been used for more than 2500 years for medicinal purposes, mostly for a variety of gynaecological conditions (3). In history, almost all VAC organs have been pharmacologically used and many contradictions have appeared with regard to VAC medicinal use even in modern times. In the late Middle Ages in Europe, the use of VAC was introduced also to celibate clerics for its supposed usefulness in reducing undesired sexual libido. After vanishing in the 19^th^ century, the pharmacological use of VAC has reemerged in Europe in the last six decades as a treatment for some gynaecological conditions such as premenstrual syndrome (PMS), cyclical mastalgia, menstrual cycle irregularities, corpus luteum insufficiency, dysfunctional uterine bleeding, and some symptoms of menopause, such as hot flushes (3–8). A recent review established that there is a role for VAC in the treatment of polycystic ovary syndrome (PCOS) (9). In fact, VAC has been approved by the German Commission E for the treatment of irregularities of the menstrual cycle, cyclical mastalgia and PMS, and such medication can be prescribed by family physicians and gynaecologists in Germany (10, 11).

As hyperprolactinaemia can occur in these gynaecological conditions, we have assessed all studies that evaluated the role of VAC on prolactin (PRL) regardless of the study outcome.

Phytotherapic drugs contain a number of pharmacological properties acting through various mechanisms and pathways. The final result is a complex set of synergistic and antagonistic interactions operating at biochemical, cellular, and physiological level (12, 13). This is particularly relevant in the context of the increasing use of complementary and alternative medicine, with current rates of use ranging between 26% and 91% (14, 15). In the USA, sales increased by 6.8% in 2014 for the eleventh consecutive year (16). An European study reported that 18.8% of the population use at least one plant supplement (17). Systematic reviews and meta-analyses on phytotherapic products have increased in number and, in the last 4 years, a considerable number of manuscripts of consistent quality assessing the clinical effectiveness of herbal remedies have been published (18). Although the majority of the publications to date have come from Western countries, there has also been an increase in interest in this area in Asiatic countries (19–22).

### Pharmacological aspects

To date, 24 distinct phytoconstituents have been isolated from the fruits of VAC, including 10 flavonoids, 5 terpenoids, 4 phenolic, 3 neolignans compounds, 1 glyceride, and 1 labdane-diterpene (23). Although the pharmacological mechanism of action of VAC has yet to be fully elucidated, its extract appears to act, at least in part, through the binding with estrogen receptors, dopamine receptors, and opiate receptors. These actions would result in changes to levels of PRL in the body, potentially leading to an improvement in the control of symptoms of menopause and PMS. More recent studies suggest a role for VAC extract in potentiating antioxidants in cancer chemoprevention and bone fracture healing. However, further research in this field is still ongoing (24–26).

Mild hyperprolactinaemia is a condition in which PRL is slightly increased and occurs for a variety of reasons, including also normal physiological response to stress in patients without a prolactinoma (**Table 1**) (27–29).

**Table 1:** Causes of hyperprolactinaemia.

Physiological
Coitus
Exercise
Lactation
Pregnancy
Sleep
Stress (incl. venipuncture)
Hypothalamic-pituitary stalk damage
Infiltrative diseases (incl. inflammatory and infective processes)
Radiations
Rathke’s cyst
Traumas with pituitary stalk section (incl. local surgery)
Tumors (i.e., craniopharyngioma, germinoma, meningioma, suprasellar extension of a pituitary tumor, hypothalamic metastases)
Pituitary diseases or dysfunctions
Acromegaly
Idiopathic
Local surgery
Lymphocytic hypophysitis
Macroadenoma with pituitary stalk compression or deafferentation
Macroprolactinaemia
Multiple hormones secreting adenoma
Prolactinoma
Traumas
Systemic conditions
Chest injuries or disorders (incl. neurogenic chest wall trauma, surgery, HZV)
Chronic renal failure
Cirrhosis
Cranial radiation
Epilepsy
PCOS
Pseudocyesis
Pharmacological
Anaesthetics
Anti-convulsants
Anti-depressants (i.e., clomipramine)
Anti-H2 receptor drugs
Anti-hypertensives
Cholinergic agonists
Cathecolamine depletors
Dopamine receptor blockers (i.e., metoclopramide, domperidone)
Dopamine synthesis inhibitors
Estrogenic unbalance (i.e., oral contraceptives, oral contraceptives withdrawal)
Neuroleptics/anti-psychotics (i.e., fluphenazine, haloperidol, paliperidone, risperidone)
Neuropeptides
Opiates and opiates antagonists (i.e, morphine, methadone)
*Adapted from Melmed et al.* (27)

PRL release from the pituitary is regulated by hypothalamic dopamine inhibition. Dopamine is secreted by the tuberoinfundibular dopaminergic neurons into the portal vessels that connect the hypothalamus with the pituitary. In the late luteal phase, women have a relative increase in serum PRL (28). Such an event is due to the decrease, to some extent, of the dopaminergic inhibition at the time of the premenstrual period (29). Increased PRL levels have been linked with mastalgia and some symptoms of PMS (30–34). One study showed that bromocriptine, a commercially available dopamine agonist, can improve breast pain and this was associated with a decrease in PRL levels (35). A study on the effects of lisuride, another dopamine agonist, demonstrated not only an improvement in symptoms of mastalgia but also on the psychological and somatic symptoms of PMS, including depression, sadness, irritability, emotional changes, and sensorial sensitivity (36). This suggests that multiple dopaminergic regions within the brain, such as the nigrostriatal and the mesolimbic system, play a role in PMS (29).

VAC exerts a central dopaminergic activity *in vitro* and *in vivo* (29, 37–42). The PRL-suppressive effect of VAC is thought to be due to a number of diterpenes, including clerodadienols, which bind to dopamine D2 receptor (37). The dopaminergic bicyclic diterpenes that have been identified are able to inhibit cAMP formation and PRL release in rat pituitary cell cultures (41). Such dopaminergic action inhibits both basal and thyrotropin releasing hormone (TRH)-stimulated PRL release (37, 38).

VAC may also bind to estrogen receptor (ER) and, as per an investigation of phytotherapy safety and efficacy in the USA, it has been evidenced that methanol extract of VAC shows significant competitive binding to ERα and ERβ (7). In addition, linoleic acid was identified as an ER ligand in VAC extracts (43). ERα or ERβ binding assays have been used to guide fractionation of VAC, which resulted in the isolation of apigenin and its identification as an ERβ-selective phytoestrogen (44). The administration of VAC extract and estradiol valerate to ovariectomized rats significantly improved learning and memory performance in both groups and the findings in each group were comparable (45). VAC extract significantly increased the expression of ERα mRNA in the hippocampus. Additionally, VAC extract at the dose of 80 mg/kg significantly increased the uterine weight in the ovariectomized rats.This result was similar to that observed in the estradiol-treated group (45). However, these data contradict the findings from a previous study, in which VAC dry extract did not induce vaginal cornification or an increase in uterine weight in rats, suggesting that it does not exert *in vivo* estrogenic properties (46).

VAC opioid activity can be beneficial in diminishing the symptoms of PMS such as depression, irritability, anxiety, mastalgia, fatigue, and headache (47, 48). Extracts of VAC have been reported to have an affinity for the opioid μ, δ, and κ receptors (MOR, DOR, and KOR, respectively). One study showed that VAC has a high affinity for MOR and KOR, and a weak affinity for DOR (39). In addition, two VAC methanol extracts have been found to activate MOR (8). Whereas a study reported an antagonist opioid activity of some flavonoids, a more recent study determined that these compounds have dose-dependent affinity for MOR and DOR resulting in the activation of these two receptors, but not in the activation of KOR (49, 50).

The possible impact of VAC on the pituitary-thyroid axis and the pituitary-adrenal axis was assessed by the administration of VAC essential oil to rats. In treated rats, the gland functional morphology showed that the relative volume density of thyrotrophic cells and thyrocytes was increased. By contrast, a decrease of pituitary corticotrophic cells associated with no changes on the adrenal tissue was observed. Consistently with the cytological findings, TSH, total thyroxine, and triiodothyronine had an increment while ACTH was lower than at the baseline and corticosterone was unaltered (51).

A possible chemopreventive activity of some VAC components (vitetrifolim D and vitexlatam) has also been hypothesized and assessed. These components may be able to stimulate NADP(H): quinone oxidoreductase type 1 (QR1) (26). QR1 is an important phase II detoxification enzyme that can protect cells against the harmful effects caused by free radicals and reactive oxygen species by catalyzing the reduction of quinones to hydroquinones. Therefore, an increased activity of the enzyme might protect cells from potential carcinogenicity (26, 52).

Additionally, VAC has been shown to exert some beneficial effects in the process of the bone healing after a long bone fracture in premenopausal women (25). Osteogenic and angiogenic factors and callus formation were assessed in a double-blind randomized placebo-controlled trial. In this study, vascular endothelial growth factor (VEGF), which is known to induce vascular invasion in fracture gap and enhance angiogenesis, was significantly increased in the VAC-treated group compared with the baseline (25, 53). Thus, phytoestrogens could exert a positive effect in the process of angiogenesis and promote the early stage of fracture healing. The VAC-treated group also showed an improved callus formation. When VAC was co-administered with magnesium, a significant elevation of osteocalcin, an osteogenic marker, has been reported (25).

### Preparations and dosage

The therapeutic effects of VAC impact on the endocrine physiology through their action on various hormones, such as PRL and female sex hormones. These effects appear to be dose-dependent. Low dose VAC resulted in lower estrogen levels and higher progesterone and PRL levels, possibly caused by the inhibition of the release of FSH and stimulation of LH (13, 54). In a study using different VAC doses, the highest VAC dose decreased the levels of PRL while FSH and LH remained unchanged (37). This dose-dependent relationship may clarify why low VAC doses stimulate breast milk production, while high doses have the opposite effect (5).

The preparations and dosage of VAC are wide-ranging. Dried, ripened, or fresh ripe fruits (berries) can be used to produce the phytotherapic medications in the form of liquid extract, dried fruit, or as a tablet or capsule. Fluid extract (40 drops daily) and tincture (35 to 45 drops, 3 times a day) have also been used (13, 55). VAC-enriched food supplements are also available. The variability in preparation and dosage was noted in the different clinical studies. Though the dosage of VAC dry extract is often 20 to 40 mg per day, in some cases higher doses (up to 1800 mg per day) were used (11, 13, 48). The appropriate dose may be best determined by the purpose for which it is being applied. For isolated mastalgia, a low VAC dosage could be appropriate (56, 57). For the treatment of PMS, a higher dosage might be necessary (58). The prescription of 4 mg/day of an extract standardized to 6% of the constituent agnuside is one of the formulations sold in the USA and this dose has been used in some studies (5).

The Committee on the Herbal Medicinal Products (HMPC), a sub-organization of the European Medicines Agency, published a final report on VAC in 2010 (59). They stated that VAC should contain the dry extract of the crude drug, which is extracted with 60% ethanol aqueous solution at the drug-extract-ratio of 6-12:1, equivalent to 180 mg of the crude herbal substance (mg/d). The specified contents of 0.05% agnuside and 0.08% casticin in the crude drug quantified by high performance liquid chromatography (HPLC) are considered to be the minimum pharmacological requirement. A medicinal product equivalent to 180 mg/d of the crude drug, as recommended by HMPC, contains at least 0.09 mg/d of agnuside and 0.144 mg/d of casticin, respectively (59). By this definition, two commercial VAC-derived drugs have been labeled for “well-established use” in 2014 and five other VAC-derived compounds have been considered as traditional products (22).

An assessment of the quality of medicinal products and health supplements that contain VAC in the European, Japanese, and American markets was published in 2014 (22). In Europe, only VAC extracts classified as drugs are commercialized. By contrast, in Japan and the USA, there are VAC-containing health foods with no such strict specifications like those of HPMC (59). The majority of health supplements assessed failed to reach the minimum standard of not less than 0.05% agnuside and 0.08% (22).

### Adverse effects, interactions, and contraindications

VAC is usually well tolerated, with infrequent, mild, and reversible adverse effects, such as nausea, headache, gastrointestinal disturbances, menstrual disorders, acne, itch, and rash (60, 61).

There are no known drug interactions with VAC but, due to its dopaminergic effects, its derivatives could interfere with certain Parkinson’s disease medications, such as dopamine agonists and dopaminergic antagonists, like metoclopramide (37, 38). Importantly, VAC has not been found to interact with oral contraceptives (OCP) and its use in treating PMS symptoms among women taking the OCP has been shown (62). However, given the significance of any interaction between OCP and VAC, this aspect should be addressed independently.

VAC is contraindicated during pregnancy and there is controversy with regard to its use during lactation. The caution is due, at least in part, to the absence of clear data and the pharmacologically varied activity of phytotherapic drugs (63, 64). Without clear evidence in terms of safety and efficacy VAC extracts will remain contraindicated during pregnancy for the general safety of mother and child (18). Similarly, VAC use during lactation is not encouraged (55).

### Objective

In this manuscript, we aimed to review and analyse the data as well as the clinical implications of the effects of VAC on PRL secretion. The use of VAC in the treatment of a number of gynaecological conditions has been quite extensively reviewed and assessed (57, 65–67). However, VAC has been proposed as a therapeutic alternative for a number of other indications, including the therapeutic management of hyperprolactinaemia. The use of VAC in the treatment of hyperprolactinaemia has not been precisely defined yet. There is a limited number of small clinical studies from which only a few concrete conclusions can be drawn. Given the explosion of the alternative remedies market, a full understanding of the physiology and pharmacology of VAC impact on PRL levels is of relevance to endocrinologists and gynaecologists and, ultimately to the patients taking these over-the-counter medications (16, 18, 67). In particular, patients with mild to moderate elevation of PRL who wish to avoid conventional medications may choose to use phytotherapic medications that appear to be safe and well tolerated, and actually may have a role in the inhibition of PRL hypersecretion (5, 37, 60, 61, 68). With regard to the administration of VAC extract to patients with prolactinoma, only a handful of case reports have been published (69, 70). Therefore, we wonder if a role for the use of VAC in the treatment of hyperprolactinaemia may finally exist.

## Methods

### Study design

We have reviewed and analysed the studies that evaluate the effects of VAC on PRL secretion published up to February 2019. We have included the studies that considered this target either directly or indirectly, thus including those studies that looked at the effect of VAC on mild hyperprolactinaemia. The studies in which VAC was assessed in association with other medication were excluded.

### Search strategy

The terms used were vitex agnus castus, agnus castus, chaste berry, and monk’s pepper both independently and cross-referenced with hyperprolactinaemia, premenstrual syndrome, polycystic ovary syndrome, and mastalgia. Additionally, further relevant papers were identified using the “related articles” function in PubMed and by exploring the reference lists of the relevant articles. We did not restrict the search to randomized controlled studies because the number of suitable papers would be extremely limited. On the other side, we have included single case reports or series.

### Data sources, study sections, and data extraction

The electronic databases explored were Pubmed, ClinicalTrials.gov, and The Cochrane Library.

## Results

### Study selection and characteristics

Altogether, we have identified a total of nine published papers. Among these, six were prospective studies (**Table 2**) (19, 54, 56, 71–73). Three published papers were case reports of patients with hyperprolactinaemia who have received treatment with a VAC extract (**Table 3**) (69, 70, 74).

**Table 2:** Prospective studies where PRL levels were assessed in patients taking a VAC extract.

Author	Study Design	Clinical Setting	Intervention	Results
Milewicz A. et al., 1993 (71)	Randomized, double-blind, placebo-controlled	37 women with menstrual cycle irregularities due to latent hyperprolactinaemia	TRH test at baseline and at the end VAC extract capsules (Strotan 20 mg/day once daily) (n=17) vs. placebo (n=20) for 3 months	Significant reduction in PRL concentration after TRH (p < 0.001) in the VAC group compared to placebo An initially shortened luteal phase normalized by 5 days Low mid-luteal progesterone levels at baseline normalized (p < 0.001 vs. placebo) Two pregnancies in VAC group
Merz P.G. et al., 1996 (54)	Placebo-controlled	Assess PRL secretion in 20 healthy male subjects during a period of 14 days	VAC extract (BP1095E1) 120 mg, 240 mg and 480 mg per day or placebo TRH test on the last day	A significant increase in the 24-hour PRL secretion profile with the lowest dose of VAC in comparison to placebo and a slight reduction with the higher dose At the end of the study, 1-hour AUC after TRH stimulation resulted in a significant PRL increase with the lowest VAC dose and a significant PRL reduction with the highest VAC dose compared to placebo
Wuttke W. et al., 1997 (56)	Placebo-controlled	101 women with cyclical mastalgia	During 3 months, 31 women received VAC (Mastodynon) solution 32 women received VAC (Mastodynon) tablets 38 enrolled in the placebo group	Compared to placebo, the basal PRL levels were significantly lower in the treatment groups Mastalgia scores were significantly lower in the treatment groups
Kilicdag E.B. et al., 2004 (72)	Prospective, randomized	40 women with mild hyperprolactinaemia (Group 1) and 40 women with cyclic mastalgia (Group 2)	In each group, patients were randomized to receive a 3-month course of either bromocriptine (Parlodel 2.5 mg twice daily) or VAC (Agnucaston 40 mg daily)	In Group 1, PRL decreased from 945.66± 173.46 to 529.19 ± 279.65 mIU/l with VAC and from 885.04 ± 177.45 to 472.68± 265.64 mIU/l with bromocriptine (normal range 25.20 – 628.53 mIU/l) In Group 2, pain scores decreased from 6.8 ± 2.29 to 1.9 ± 1.92 with VAC and from 6.3 ± 2.45 to 0.89 ± 1.05 with bromocriptine In both groups, p < 0.01 comparing pre- and post-treatment and no difference between VAC and bromocriptine treatment
Ma L. et al., 2010 (19)	Prospective, randomized, double-blind, placebo-controlled	67 women with PMS	Women received either VAC (BNO 1095 40mg/day) for 3 months or placebo	Significant decrease on percentages of all PMS symptom scores in the treatment group compared to placebo, except lower abdominal cramping No significant decrease on serum PRL at the third treatment cycle compared to baseline
Shahnazi M. et al., 2016 (73)	Randomized, triple-blind, controlled trial	70 women with PCOS	Women were randomized to take either VAC or low-dose oral contraceptives (LD) for 3 months	Significant decrease in DHEA-S in both groups Normalization of menstrual cycle duration in about 68.6% of LD group and in 60% of VAC group No significant difference in PRL and free testosterone before and after intervention in both groups

**Table 3:** Case reports of patients with hyperprolactinaemia who have been treated with a VAC extract.

Author	Clinical setting	Intervention	Results
Meczekalski B. et al., 2015 (74)	A 27-year-old woman with hyperprolactinaemia (40.2 ng/ml, normal value 5-25 ng/ml) and oligomenorrhea for 1 year	Patient intolerant to bromocriptine, treated with VAC (Cyclodynon 40 mg/day) for 2 months	PRL reduction to 8.7 ng/ml, normalization of the menstrual cycle
Gallagher J. et al., 2008 (70)	An 18-year-old woman presented to a women’s health clinic with a 2-year history of oligomenorrhoea and a 9-month history of amenorrhea; galactorrhoea on examination; no macroprolactin High PRL 2166 IU/l (reference value: 80–600 IU/l)	Patient referred to the endocrinology service, where was seen 6 months later In the meantime, VAC (Agnolyt 15 drops/day) was prescribed for 3 months by a complementary health practitioner, possibly for treating a skin condition	On review at the endocrinology clinic, patient reported a regular 28-day cycle for 3 months prior to the appointment MRI showed a 2 mm microadenoma Advised to switch to cabergoline
Tamagno G. et al., 2007 (69)	A 31-year-old woman with amenorrhea and galactorrhoea Basal PRL 2207 mU/l (reference values: 50–600 mU/l) MRI showed a 5mm cystic pituitary microadenoma	Refused conventional D2 agonist drugs Started on VAC 20 drops twice daily for 3 months	PRL decreased to 1766 mU/l after 3 months with VAC 3 months after stopping VAC, PRL was 1609 mU/l and MRI remained unchanged

### Synthesis of the findings

Though there is some evidence suggesting a role of VAC in the management of hyperprolactinaemia in selected patients, the lack of large randomized controlled trials and the still unclear pharmacological aspects through which VAC extract might work affect the possibility of reaching solid enough conclusions. Unfortunately, the variety of the methodological aspects and of the design of the studies prevent from drawing any reproducible conclusions. However, it appears that VAC can be able to impact on PRL secretion in selected patients, especially those with only mild hyperprolactinaemia, and in general terms seems to be a safe and well tolerated phytotherapic agent.

## Discussion

### Main findings

Hyperprolactinaemia is a relatively common condition in the female population. In addition, the popularity of non-conventional medications, including phytotherapic drugs, has progressively increased in the recent years. VAC has been proposed as a phytotherapic medication with a potential to reduce PRL levels *in vivo*. Against the beliefs held by monks in the middle ages, VAC may actually have a role in increasing, as opposed to decreasing, male libido. The available literature on the effect of VAC, although limited in number, suggests a potential role of VAC in the treatment of mild hyperprolactinaemia. All but two of the published studies included in our data analysis report a decrease of the PRL levels as a result of the use of VAC extracts. One of the studies that failed to demonstrate a decrease in PRL did not provide reference ranges for PRL, stating only that it did not significantly change, and the results are therefore difficult to draw conclusions from (19). In the other manuscript that found no significant changes on PRL levels after three cycles of treatment with VAC, the authors stated that the result might be explained by the different methods used for measuring the PRL levels (ELISA, versus specific chemiluminescence assay method) (71, 73). Moreover, VAC effects might be dose-dependent and also this aspect can contribute to the heterogeneity of the results among the various studies. For example, a low VAC dose has been found to increase PRL while, in the same study, a higher dose decreased the PRL levels (54). This observation may highlight further the complicated pharmacology of the active constituents of VAC.

In our opinion, the systematic characterization and assessment of the single molecular constituents of VAC extract is the only possible way for discriminating which of them, alone or in combination, are able to exert a pharmacological effect on PRL secretion and, therefore, on the circulating PRL levels. However, we understand that such an approach would require a significant financial investment and a heavy dedicated workload for the researchers. Possibly these practical aspects have contributed to the limited research carried out on this topic to date. In fact, we shall keep in mind that there is already a good number of well-established, effective, and reasonably safe conventional medications available on the market (cabergoline and bromocriptine, for citing the two most widely used dopamine agonists in the treatment of hyperprolactinaemia). A comparison of VAC and bromocriptine use on PRL levels in women with mild hyperprolactinaemia found a comparable reduction in both groups (72). Although arising from a small study, these findings are interesting and warrant further evaluation. The reduction of PRL after VAC administration has been demonstrated elsewhere, including a decrease in TRH-stimulated PRL release and among women with mastalgia (56, 71).

Unfortunately, the assessment of a possible correlation between the changes of the PRL levels, when documented, and the changes of the signs and symptoms reported by the patients enrolled in the various studies cannot be performed in a valid way considering the inhomogeneous collection of data and observations from study to study.

In addition to the few studies where the changes of specific symptoms and signs were assessed, some case reports described a reduction in the symptoms of hyperprolactinaemia, such as galactorrhea and menstrual cycle irregularities, in women taking VAC. Interestingly, in two of these case reports, the patient had a pituitary microadenoma (69, 70).

### Limitations

Unfortunately, the small number of studies and case reports published to date and included in this analysis of the published clinical data are significantly heterogeneous. Many of these works lack a robust enough methodology, often with a small number of enrolled patients and with a short duration of the treatment and/or observation time. Moreover, the different types of VAC extract and doses used in the reported studies inevitably impact in a significant way on the possibility of carrying out a solid and reliable analysis of the results. There are also some obvious difficulties usually encountered by the researchers when studying the physiological levels of PRL *in vivo*. For example, physiological stressors, such as venipuncture, may impact on the findings and should also be accounted for in any study design. A quite recent meta-analysis focusing on the VAC in PMS highlighted the paucity of well-structured studies (66). Moreover, there is a generic risk of selection bias associated with the meta-analyses. The poor homogeneity and the paucity of the published data unfortunately prevents from a statistical analysis of the published findings.

Therefore, it is not possible to reach any solid conclusions on the potential role of VAC extract in the treatment of hyperprolactinaemia. Additionally, a direct link between VAC and decreased PRL levels has been not undoubtedly identified yet.

We think that further investigations, both *in vitro* and *in vivo* and including also more stringently designed studies and high quality trials, should be encouraged on the basis of the currently available data from the literature.

However, for the moment the possible pharmacological use of VAC extractin patients with hyperprolactinaemia should remain strictly limited to the few patients who are looking for non-conventional forms of treatment and, in our opinion, also in these cases the well-established dopamine agonists should be initially offered as first-line treatment and, then, re-offered during the follow-up within a reasonable observation time, if no evidence of satisfactory therapeutic effects will be achieved with VAC.

The precise characterization of one or more VAC extract molecules able to therapeutically impact on the PRL levels and proving that such VAC component or components are effective and safe *in vivo* is, in our opinion, the only option for possibly considering the use of VAC, in the form of specific component or components, for the treatment of hyperprolactinaemia. Probably, the well-established presence on the market of valuable and reliable dopamine agonists contributes to leave a very little room only to non-conventional alternatives in the pharmacological armamentarium of the treatment of hyperprolactinaemia.

### Conclusions

The interest in phytotherapic drugs has lately increased worldwide, both among patients and in the scientific community. VAC is a well-known phytotherapic drug with a relatively proven role in some gynaecological conditions and, in particular, in the treatment of PMS. The efficacy of VAC extracts in the treatment of other conditions, like hyperprolactinaemia, is still a matter of debate. There are no published studies which produce unequivocal evidence of VAC efficacy in this clinical setting and, indeed, the complexity of the physiological, pharmacological, and clinical aspects of VAC and hyperprolactinaemia does not help. Even in this context affected by a significant degree of uncertainty, the available laboratory and clinical findings seem to support the existence of some effects of VAC on PRL secretion with a possible therapeutic impact in selected patients. Larger and higher quality studies are required for exploring the VAC effects on hyperprolactinaemia and, possibly, confirming the hypothesis of a role of this phytotherapic medication in such a clinical setting. Some important questions, for example related to the therapeutic VAC dosage and the target patient population, are still unanswered. However, we think that VAC might represent a potentially reasonable and safe phytotherapic option for the management of selected patients with mild hyperprolactinaemia who wish to be treated with phytotherapy.

## Author contributions

LP: Data curation, Formal analysis, Investigation, Methodology, Resources, Validation, Visualization, Writing – original draft. JL: Data curation, Resources, Validation, Visualization, Writing – review & editing. GT: Conceptualization, Data curation, Formal analysis, Investigation, Methodology, Project administration, Resources, Supervision, Validation, Visualization, Writing – original draft, Writing – review & editing.
